# Association Between EEG Patterns and Serum Neurofilament Light After Cardiac Arrest: A Post Hoc Analysis of the TTM Trial

**DOI:** 10.1212/WNL.0000000000200335

**Published:** 2022-06-14

**Authors:** Linnéa Grindegård, Tobias Cronberg, Sofia Backman, Kaj Blennow, Josef Dankiewicz, Hans Friberg, Christian Hassager, Janneke Horn, Troels W. Kjaer, Jesper Kjaergaard, Michael Kuiper, Niklas Mattsson-Carlgren, Niklas Nielsen, Anne-Fleur van Rootselaar, Andrea O. Rossetti, Pascal Stammet, Susann Ullén, Henrik Zetterberg, Erik Westhall, Marion Moseby-Knappe

**Affiliations:** From Neurology (L.G., T.C., N.M.-C., M.M.-K.), Clinical Neurophysiology (S.B., E.W.), Cardiology (J.D.), and Anaesthesia and Intensive Care (H.F.), Department of Clinical Sciences Lund, Lund University, Skåne University Hospital, Malmö; Department of Psychiatry and Neurochemistry (K.B., H.Z.), Institute of Neuroscience and Physiology, the Sahlgrenska Academy, University of Gothenburg; Clinical Neurochemistry Laboratory (K.B., H.Z.), Sahlgrenska University Hospital, Mölndal, Sweden; Department of Cardiology (C.H.), Rigshospitalet and Department of Clinical Medicine, University of Copenhagen, Denmark; Departments of Intensive Care (J.H.) and Neurology/Clinical Neurophysiology (A.-F-V.R.), Amsterdam Neuroscience, Amsterdam UMC, Academic Medical Center, University of Amsterdam, the Netherlands; Departments of Clinical Neurophysiology (T.W.K.) and Cardiology (J.K.), Rigshospitalet University Hospital, Copenhagen, Denmark; Department of Intensive Care (M.K.), Medical Center Leeuwarden, the Netherlands; Clinical Memory Research Unit, Faculty of Medicine (N.M.-C.), and Wallenberg Centre for Molecular Medicine (N.M.-C.), Lund University; Anaesthesia and Intensive Care, Department of Clinical Sciences Lund (N.N.), Lund University, Helsingborg Hospital, Sweden; Department of Neurology (A.O.R.), CHUV and University of Lausanne, Switzerland; Department of Anesthesia and Intensive Care (P.S.), Centre Hospitalier de Luxembourg; Department of Life Sciences and Medicine (P.S.), Faculty of Science, Technology and Medicine, University of Luxembourg; Clinical Studies Sweden (S.U.), Skåne University Hospital, Lund; Department of Neurodegenerative Disease (H.Z.), UCL Institute of Neurology; UK Dementia Research Institute at UCL (H.Z.), London, UK; and Hong Kong Center for Neurodegenerative Diseases (H.Z.), China.

## Abstract

**Background and Objectives:**

EEG is widely used for prediction of neurologic outcome after cardiac arrest. To better understand the relationship between EEG and neuronal injury, we explored the association between EEG and neurofilament light (NfL) as a marker of neuroaxonal injury, evaluated whether highly malignant EEG patterns are reflected by high NfL levels, and explored the association of EEG backgrounds and EEG discharges with NfL.

**Methods:**

We performed a post hoc analysis of the Target Temperature Management After Out-of-Hospital Cardiac Arrest trial. Routine EEGs were prospectively performed after the temperature intervention ≥36 hours postarrest. Patients who awoke or died prior to 36 hours postarrest were excluded. EEG experts blinded to clinical information classified EEG background, amount of discharges, and highly malignant EEG patterns according to the standardized American Clinical Neurophysiology Society terminology. Prospectively collected serum samples were analyzed for NfL after trial completion. The highest available concentration at 48 or 72 hours postarrest was used.

**Results:**

A total of 262/939 patients with EEG and NfL data were included. Patients with highly malignant EEG patterns had 2.9 times higher NfL levels than patients with malignant patterns and NfL levels were 13 times higher in patients with malignant patterns than those with benign patterns (95% CI 1.4–6.1 and 6.5–26.2, respectively; effect size 0.47; *p* < 0.001). Both background and the amount of discharges were independently strongly associated with NfL levels (*p* < 0.001). The EEG background had a stronger association with NfL levels than EEG discharges (R^2^ = 0.30 and R^2^ = 0.10, respectively). NfL levels in patients with a continuous background were lower than for any other background (95% CI for discontinuous, burst-suppression, and suppression, respectively: 2.26–18.06, 3.91–41.71, and 5.74–41.74; effect size 0.30; *p* < 0.001 for all). NfL levels did not differ between suppression and burst suppression. Superimposed discharges were only associated with higher NfL levels if the EEG background was continuous.

**Discussion:**

Benign, malignant, and highly malignant EEG patterns reflect the extent of brain injury as measured by NfL in serum. The extent of brain injury is more strongly related to the EEG background than superimposed discharges. Combining EEG and NfL may be useful to better identify patients misclassified by single methods.

**Trial Registration Information:**

ClinicalTrials.gov NCT01020916.

EEG is the most commonly used method for predicting neurologic outcome after cardiac arrest (CA).^1^ Within seconds of circulatory arrest, the EEG becomes suppressed and after return of circulation, if recovery occurs, neuronal activity progresses gradually from a suppressed to a more continuous background.^2,3^ The specific time point of EEG examination is therefore considered crucial for its prognostic relevance. An early recovery of a continuous and normal voltage background within the first 12–24 hours is associated with good neurologic outcome.^4-6^

A classification of EEG patterns into benign, malignant, and highly malignant was proposed based on the terminology from the American Clinical Neurophysiology Society (ACNS).^7,8^ This classification concerns routine EEGs obtained after the first 36 hours postarrest. It has been externally validated and found to predict neurologic outcome after CA with high specificity and substantial interrater reliability.^4,9,10^ Two background patterns are considered highly malignant; suppression (all EEG activity <10 μV) and burst-suppression (suppression periods alternate with bursts of cortical activity).^7,10^ Either of these background patterns ≥24 hours after CA, regardless of the presence of discharges, is considered a strong predictor of poor neurologic outcome according to recent guidelines.^11,12^

The presence of abundant rhythmic/periodic discharges is considered a criterion of a malignant EEG and outcome is often poor.^7,10^ Discharges may reflect severe ischemic damage and antiepileptic treatment might not improve outcome.^13^ In a number of patients, however, neuronal injury is less extensive and anticonvulsant treatment may be beneficial.^14^

Studies for prediction of neurologic outcome often permit clinical decision-making based on the results from the same examinations, and the risk of self-fulfilling prophecies cannot be excluded. In contrast, highly sensitive blood biomarkers for brain injury have been used as surrogate markers in clinical studies.^15,16^ Brain injury markers have the advantage of being objective and quantitative indicators, especially if not available upon clinical decision-making. The most accurate blood biomarker of brain injury after CA described to date is the neuroaxonal injury marker neurofilament light (NfL), which is superior to S100B, neuron-specific enolase (NSE), and tau.^17,18^ Analysis of NfL is not standardized nor do validated cutoff values exist for its use in neuroprognostication after CA. Nonetheless, using an early quantitative measure such as NfL levels as a surrogate marker for neuroaxonal injury could give unique insights into whether EEG abnormality is directly associated with brain injury and not only neurologic outcome, which may be biased by self-fulfilling prophecies.

We hypothesized that the hierarchy of benign, malignant, and highly malignant EEG patterns is reflected by increasing NfL levels as a measure of acute brain injury after CA. We further explored associations between NfL and 2 fundamental elements of the EEG: background and superimposed discharges. Based on the prognostic accuracies of the highly malignant pattern to predict poor outcome after CA, we hypothesized that the EEG background is more strongly associated with brain injury than EEG discharges.

## Methods

### Patients

This is a post hoc analysis of the Target Temperature Management After Out-of-Hospital Cardiac Arrest trial (TTM-trial), an international multicenter trial randomizing patients ≥18 years of age with CA of a presumed cardiac origin to a temperature control intervention of either 33°C or 36°C, as previously published (clinicaltrials.gov; NCT01020916).^19,20^ A total of 939 patients were included in the modified intention-to-treat population.^19^ Detailed information on neurologic prognostication and decisions on level of care have been reported previously.^21-23^ Neurologic outcome was assessed at 6 months postarrest according to the Cerebral Performance Category (CPC) Scale. Poor outcome was defined as CPC 3–5 (severe cerebral disability, vegetative state, or death). The presence of any clinical seizures was prospectively documented by the treating physician.^24^

### Standard Protocol Approvals, Registrations, and Patient Consents

The protocol of the trial was approved by the ethics committees in the participating countries in accordance with national requirements and the principles of the Declaration of Helsinki.^19,25,26^ A list of participating sites and ethics approvals for each country has been published.^19^ Inclusion in the study was considered an emergency procedure due to the time-sensitive intervention studied. Participants in the study were per eligibility criteria unconscious at the time of inclusion and therefore could not consent to inclusion. Consent from a legal surrogate was obtained as soon as possible or was waived (next of kin only informed about the trial) as per decision of the ethics committees in each country. Written informed consent was obtained as soon as possible from all participants who regained mental capacity.^19^

### NfL Biomarker Measurements

Within TTM-trial, 29/36 trial sites prospectively collected serum samples at 24, 48, and 72 hours after return of spontaneous circulation (ROSC), which were subsequently frozen and stored in a central biobank.^27^ In this study, the highest level of NfL at 48 or 72 hours (peak NfL) was used for the analyses because median NfL concentrations were twice as high at 48–72 hours postarrest compared with 24 hours.^17^ Serum NfL concentrations were measured using an in-house ultrasensitive Single molecule array (Simoa) assay on an HD-1 Analyzer (Quanterix). The measurements were performed after trial completion in 1 round of experiments using 1 batch of reagents by board-certified laboratory technicians who were blinded to clinical data.^17^ The results were not part of the prognostication algorithm used to guide patient management.

### EEG Procedures

Full montage routine EEGs were prospectively recorded in patients who were still comatose after the 36 hours temperature control intervention as previously reported and a local interpretation was available to the treating physician.^7,10,28^ EEG was mandated as part of the neurologic evaluation in patients still unconscious after the temperature intervention. Patients who died or were awake and following commands prior to neuroprognostication were excluded from this study. After study completion, EEGs were collected to a central database and reviewed systematically by blinded investigators. EEG patterns were classified according to the grading system described by Westhall et al.^7^ using the standardized ACNS terminology.^7,8,10^

- Benign EEG–continuous or nearly continuous background without malignant features is described below
- Malignant EEG—discontinuous background, reversed anterio-posterior gradient or low-voltage background, abundant (≥50% of the recording) rhythmic or periodic discharges or unequivocal seizures
- Highly malignant EEG—burst-suppression or suppression background with or without discharges

Background activity was categorized into 4 groups according to continuity:

- Continuous or nearly continuous background (suppression periods <10%)
- Discontinuous background (suppression periods 10–<50%)
- Burst-suppression background (suppression periods ≥50%)
- Suppressed background (all background activity suppressed <10 μV)

The amount of electrographic discharges was categorized into 3 groups:

- None/rare—no discharges or rare rhythmic/periodic discharges (<1% of the recording) or sporadic epileptiform discharges <1/10 seconds
- Intermediate—occasional to frequent rhythmic/periodic discharges (1–<50% of the recording) or sporadic epileptiform discharges >1/10 seconds
- Abundant—abundant to continuous rhythmic/periodic discharges (≥50% of the recording) or at least 1 unequivocal electrographic seizure

### Statistical Analysis

Variables are presented as numbers, percentages, and median values where applicable. For all statistical analyses, peak NfL values were log_10_-transformed. One-way analysis of variance (ANOVA) was used for the tests of associations between NfL levels and different EEG features. *t* Tests and 1-way ANOVA with a Tukey honestly significant difference was used for the between-group analyses. To assess the association between EEG background and discharges with the NfL levels, we used a 2-way ANOVA. The effect measures presented are the mean difference of log_10_ NfL between the groups transformed back to the original scale demonstrating the multiplicative difference between the groups. A *p* value of <0.05 was considered significant. We used R version 3.6.0 and SPSS Statistics version 25 for the statistical analyses.

### Data availability

Anonymized data not published within this article may be shared at the request of any qualified investigator for purposes of replicating procedures and results.

## Results

### Patients

**Figure 1:**
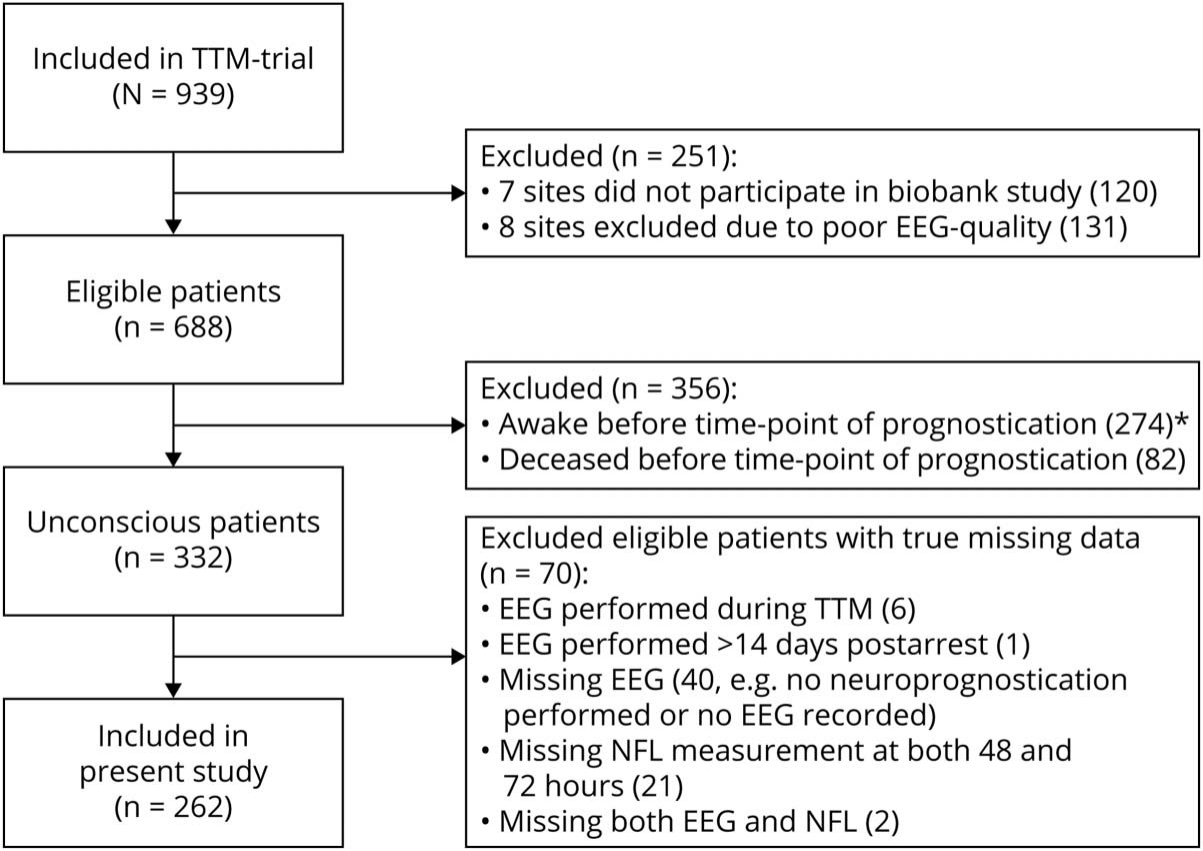
Flow Chart of Patient Inclusion The modified intention-to-treat population in the Target Temperature Management After Out-of-Hospital Cardiac Arrest trial (TTM-trial) consisted of 939 patients. Nine study sites were excluded due to technical issues in providing EEGs for export in sufficient quality required for centralized evaluation. EEG: routine EEG performed after rewarming but <14 days postarrest. *21/274 (7.6%) patients who were awake prior to prognostication still had poor neurologic outcome at 6 months. NfL = neurofilament light.

We included all patients who were still unconscious at 36 hours postarrest who had a routine EEG after rewarming and at least 1 NfL measurement at 48 or 72 hours (n = 262) (Figure 1). Included patients had longer duration from CA to ROSC, longer hospital stays, more often a poor neurologic outcome, higher NfL levels, and withdrawal of life-sustaining therapy (WLST) were more often performed compared with patients excluded from the study (Table 1). Eligible patients with true missing data (n = 70) had similar characteristics as included patients.

**Table 1:**
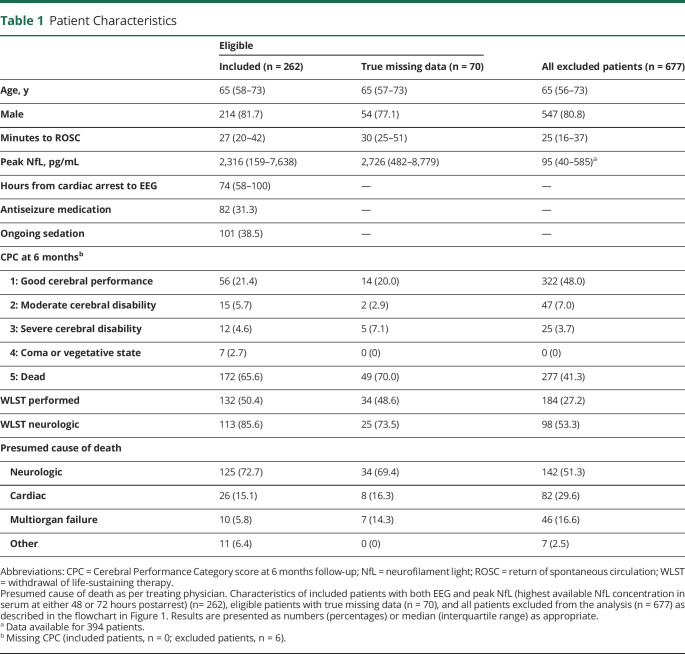
Patient Characteristics

EEG recordings had a minimum duration of 10 minutes with a median time from CA to EEG of 74 hours (interquartile range 58–100 hours). During EEG registration, 101/262 (39%) patients had ongoing sedation, of whom 67/101 (66%) had no electrographic discharges.

### NfL in Highly Malignant, Malignant, and Benign EEG Patterns

**Figure 2:**
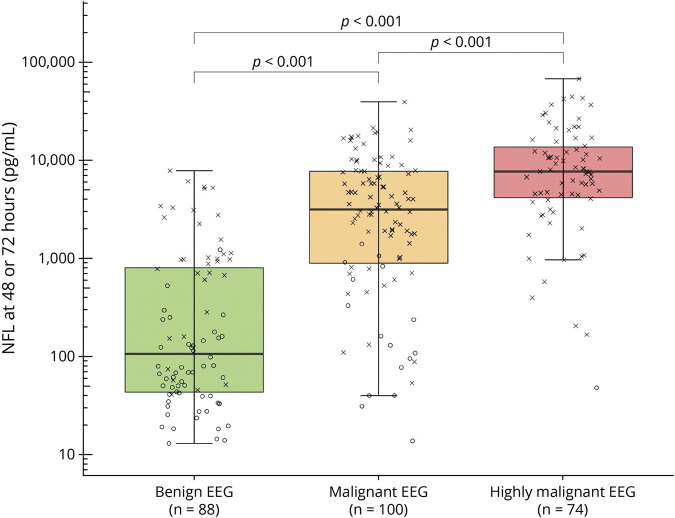
Highly Malignant, Malignant, and Benign EEG Patterns and Serum NfL Boxplot demonstrating logarithmic peak neurofilament light (NfL) levels (highest serum neurofilament levels at either 48 or 72 hours postarrest) for EEG patterns as defined by Westhall et al.^7^: “highly malignant”: burst-suppression or suppression with or without discharges; “malignant”: discontinuous, reversed anterio-posterior gradient or low-voltage background, abundant rhythmic or periodic discharges or unequivocal seizures; “benign”: continuous background without malignant features. Neurologic outcome for each patient is indicated through “X” (poor outcome, Cerebral Performance Category [CPC] 3–5) or “O” (good outcome, CPC 1–2) at 6 months’ follow-up. Peak NfL was increasingly higher in more malignant EEG patterns (*p* < 0.001).

Twenty-eight percent of patients fulfilled criteria of a highly malignant EEG pattern, 38% had a malignant EEG, and 34% had a benign EEG with no malignant features (Table 2). NfL levels were 2.9 times higher in patients with highly malignant compared with malignant EEG patterns (95% CI 1.4–6.1, *p* < 0.001) and 13 times higher in patients with malignant compared with benign EEG patterns (95% CI 6.5–26.2, *p* < 0.001; effect size 0.47) (Figure 2). When categorizing EEGs, 20 patients with a malignant pattern were classified to have a continuous background without discharges due to a reversed anterio-posterior gradient, low voltage, or a combination of both. Furthermore, 13 patients with a benign pattern were classified to have a continuous background with intermediate discharges because the amount of discharges did not fulfill the criteria for a malignant pattern. WLST due to neurologic reasons was performed in 8/88 (9%) patients with benign, 55/100 (55%) patients with malignant, and 50/74 (68%) patients with highly malignant EEG patterns.

**Table 2:**
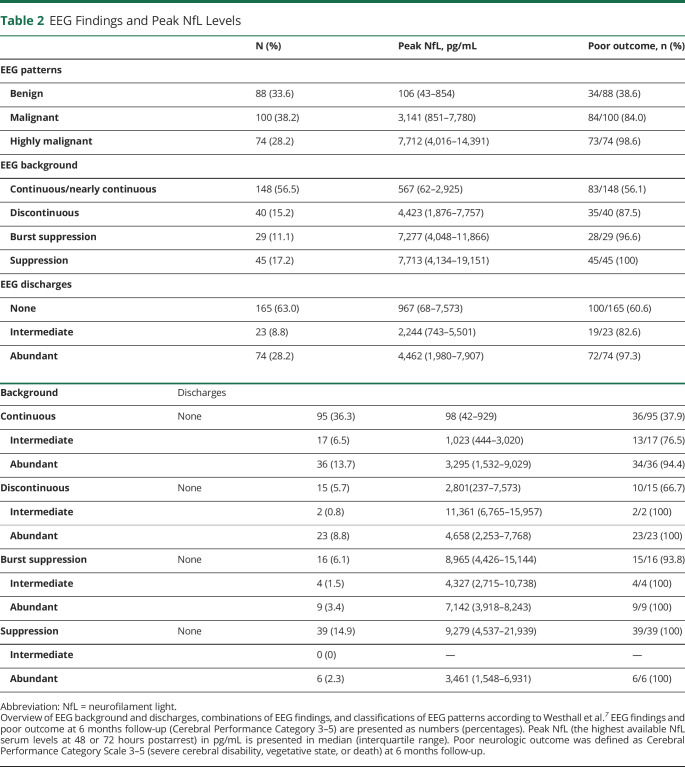
EEG Findings and Peak NfL Levels

### EEG Background Continuity and NfL

**Figure 3:**
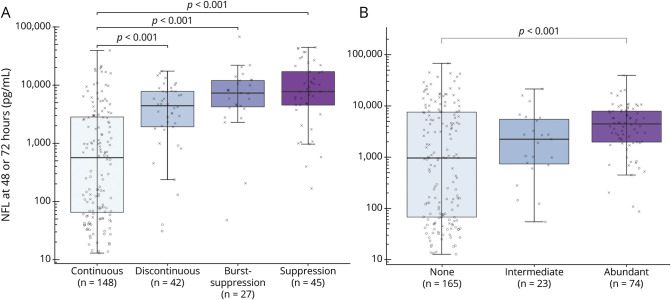
EEG Background, Discharges, and Serum NfL Boxplots demonstrating logarithmic peak neurofilament light (NfL) according to EEG background (A) or the presence of discharges (B). Neurologic outcome for each patient is indicated through “X” (poor outcome, Cerebral Performance Category [CPC] 3–5) or “O” (good outcome, CPC 1–2) at 6 months’ follow-up. The highest peak NfL levels were seen in patients with burst-suppression or suppression. In patients with a continuous background, patients with poor outcome had higher NfL levels than did patients with good outcome (median 60.7 [interquartile range (IQR) 32.8–118.7] pg/mL vs median 998.3 [IQR 366.0–3,449.7] pg/mL; *p* < 0.005). Peak NfL was higher in patients with an abundant amount of discharges than in patients without discharges (*p* < 0.001). In patients without discharges, NfL levels were higher in patients with poor outcome than in patients with good outcome (median 60.7 [IQR 32.9–128.4] pg/mL vs median 5,305.5 [IQR 1,064.8–12,926.6] pg/mL; *p* < 0.005).

EEG background was continuous in 57%, discontinuous in 15%, burst-suppression in 11%, and suppression in 17% (Table 2). NfL concentrations for patients with a continuous background were lower than for any other background (discontinuous, 95% CI 2.26–18.06; burst-suppression, 95% CI 3.91–41.71; and suppression, 95% CI 5.74–41.74; effect size: 0.30, *p* < 0.001 for all) (Figure 3A). NfL levels did not differ between discontinuous and burst-suppression background (*p* = 0.42) or between burst-suppression and suppressed background (*p* = 0.97).

### Electrographic Discharges and NfL

Most EEGs (63%) were classified as without discharges; 9% had an intermediate amount and 28% had abundant discharges (Table 2). NfL levels were higher in patients with abundant discharges than in patients without discharges (*p* < 0.001) (Figure 3B). Overall, 24% of patients had ongoing antiseizure medication during EEG recordings (Table 1). Only 27/165 (16%) patients without electrographic discharges were treated with antiseizure medication, in contrast to 11/23 (48%) with an intermediate amount and 44/74 (59%) patients with abundant discharges. We found that 127/262 (48%) patients had any type of clinical seizures during the first 3 days after CA or during the EEG recording. Of these 127 patients, 53 (42%) had no discharges, 14 (11%) had an intermediate amount of discharges, and 60 (47%) had abundant discharges on the EEG. When excluding patients with clinical seizures from our model, NfL levels remained higher in patients with abundant discharges than in patients without discharges (*p* = 0.002; data not shown).

### NfL When Combining EEG Background and Discharges

**Figure 4:**
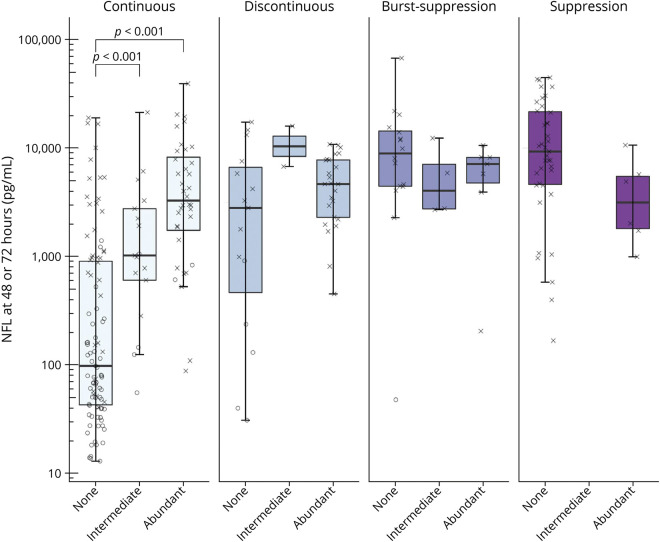
EEG Background, Superimposed Discharges, and Serum NfL Boxplot demonstrating logarithmic peak neurofilament light (NfL) in patients with continuous, discontinuous, burst-suppression, and suppressed EEG background and the presence of superimposed discharges. Neurologic outcome for each patient is indicated through “X” (poor outcome, Cerebral Performance Category [CPC] 3–5) or “O” (good outcome, CPC 1–2) at 6 months’ follow-up. In patients with a continuous background, NfL levels were higher in patients with an intermediate or abundant amount of discharges than in patients without discharges (*p* < 0.001). No other differences could be found between noncontinuous backgrounds and the presence of discharges.

When evaluating the EEG background and the presence of discharges together, both the background (*p* < 0.001) and discharges (*p* < 0.001) were independently associated with NfL levels (Figure 4), the background explaining a larger proportion of variance than discharges (R^2^ = 0.30 and R^2^ = 0.10, respectively). Discharges were associated with higher NfL levels only in the group of patients with a continuous EEG background (*p* < 0.001). Hence, when the background was burst-suppression or suppression, there was no difference in NfL levels among patients with discharges compared with patients without discharges (*p* = 0.43 and *p* = 0.06, respectively). Notably, no patient with a discontinuous background survived to 6-month follow-up if discharges were present, and NfL levels in patients with this combination were high.

## Discussion

We confirmed that the standardized classification of post-CA EEGs into benign, malignant, and highly malignant patterns^7^ is reflected by increasing NfL concentrations, indicating the degree of neuroaxonal injury. EEG background and electrographic discharges at 36 hours or later postarrest were independently associated with blood NfL levels, where the background had the stronger association with NfL.

The highly malignant EEG pattern, which includes any background with ≥50% suppression, has very high specificity for prediction of poor neurologic outcome.^11^ Substantially elevated NfL levels in most of these patients quantitatively confirm that highly malignant backgrounds are indeed associated with extensive neuroaxonal injury.^7,10^

Benign EEG patterns ≥36 hours postarrest indicate a good prognosis, whereas malignant EEG patterns represent an intermediate group with both good and poor clinical outcomes.^7,10,11^ In common with other classifications of EEG patterns, the classification that we used includes combinations of background patterns and superimposed discharges. To better understand which elements are more related to the extent of neuronal injury, we chose to evaluate EEG background and discharges separately.

Background activity was divided into 4 groups according to the amount of suppression periods. We did not detect any difference in NfL levels between suppressed and burst-suppression backgrounds. Both backgrounds could have different pathophysiologic correlates but demonstrate similar degrees of neuroaxonal injury, where a suppressed background may indicate severe functional damage to pyramidal cells or interneurons and burst-suppression a severely damaged cortex with less affected subcortical areas.^29-31^ A histopathologic study found severe hypoxic-ischemic encephalopathy in 96% of patients with suppression or burst-suppression backgrounds.^29^ On a group level, suppression of EEG background through sedatives has not been found to influence the reliability of poor outcome prediction; nonetheless, false-positive cases have been reported.^7,10,32^ In our cohort, 1 patient had a good outcome despite a burst-suppression background, presumably due to ongoing significant sedation, as previously reported*.*^10^ The peak NfL concentration in this patient was within age-dependent normal values of our laboratory, illustrating how the combination of EEG and a quantitative biomarker may reduce the risk of falsely pessimistic predictions.^33^

In patients with a discontinuous background, we found a broad range of NfL levels, indicating that an isolated finding of a discontinuous background does not reliably indicate severe brain injury. This is in accordance with the 2021 guidelines of the European Resuscitation Council and the European Society of Intensive Care Medicine stating that a discontinuous background has low prognostic performance within the first 24 hours postarrest and inconsistent performance thereafter.^12^

The lowest NfL levels in our cohort were found in patients with a continuous EEG background. However, there were also a substantial amount of patients with poor outcome with highly elevated levels of NfL in this group. An early return of a continuous background within the first 12–24 hours postarrest is often predictive of a good neurologic outcome.^34,35^ In analogy with brainstem reflexes, a continuous background may recover later than 24 hours postarrest despite extensive brain injury and is therefore not automatically predictive of good outcome.^4,23^ At relatively long latency after arrest, this pattern could still be associated with severe histopathologic brain damage.^29^ Whether the patients in our cohort restored a continuous background late cannot be determined, as continuous EEG monitoring was not used. We hypothesize that this may have been the case in patients with poor outcome with severely elevated NfL levels. Furthermore, a continuous background can also display malignant features such as a reversed anterio-posterior gradient.^7^

The presence of abundant discharges (>50% of the recording) is also a criterion for a malignant EEG and indicates a poor prognosis.^7,10^ We found that when evaluating discharges irrespective of EEG background, patients with an abundant load had higher median NfL concentrations than patients without discharges. The group of patients with an intermediate amount of discharges did not differ in NfL levels compared with the other 2 groups. In another substudy from the TTM-trial using continuous EEG monitoring with a reduced montage, NfL levels were elevated at 72 hours postarrest in patients with electrographic status epilepticus compared with patients without.^36^ Another study compared EEG and NSE and found no difference in levels between patients with or without discharges, possibly due to lack of separation between different amounts of discharges.^37^ Electrographic discharges are considered a sign of increased excitatory activity of the pyramidal cells due to the loss of inhibitory interneurons, caused by either severe encephalopathy or injury to specific brain areas only.^38^ On neuroimaging, discharges can be associated with both cortical and subcortical lesions, but MRI was reported as normal in 20% of patients with generalized periodic discharges.^39^ In a proposed model, a 5% reduction in cortical disinhibition was sufficient to induce generalized periodic discharges.^40^ Whether normal EEG patterns can be reestablished may depend on the extent of injury to cortical networks and reversibility of synaptic failure.^38^ It is unclear whether discharges induce additional injury through excitotoxicity or if they are solely the result of the hypoxic-ischemic injury caused by the arrest.^11^

Discharges on a burst-suppression or suppressed background were not associated with a change in NfL levels in our study. A recent study with 7 patients also described higher NfL levels in patients with a suppressed background than in patients with generalized periodic discharges.^41^ A small histopathologic study reported similar findings.^29^ This supports the assumption that neurons unable to produce either background activity or discharges are more injured than neurons generating them.

In our study, NfL levels did not differ whether discharges were present or not on a discontinuous background. Nonetheless, no patient with a discontinuous background survived to 6-month follow-up if discharges were present and NfL levels in patients with this combination were high.

In cases with a continuous background, NfL was higher in those with discharges than without. Our data imply that discharges may only provide additional prognostic information when superimposed on a continuous or discontinuous background and that they are not necessarily associated with poor outcome. Previous studies after CA report that in patients with good outcome, status epilepticus evolved from a continuous background.^13,42^ The design of our study cannot confirm whether discharges cause further NfL elevation in addition to the primary hypoxic-ischemic injury. Further research is required to identify those patients where anticonvulsant treatment may improve outcome and repeated NfL sampling at later time points could be valuable. We recently reported that low levels of brain injury markers are predictors of a favorable neurologic outcome, indicating that EEG patterns and biomarkers together may help guide clinical decision-making.^43^

Strengths of our study include the international multicenter design, a conservative approach to neurologic prognostication, and strict criteria for WLST.^19,44^ As previously published, serum samples were prospectively collected from all patients at sites participating in the biobank substudy and the number of missing samples was low.^17^ In contrast to the guideline-recommended biomarker NSE, which is also present in erythrocytes and neuroendocrine tumors, NfL levels are not falsely elevated in the presence of hemolysis.^17,27^

The TTM-trial included adult patients with a presumed cardiac cause of arrest, and because we evaluated the extent of brain injury, we suspect that results would be similar in patients with anoxic brain injuries due to other causes. However, our results should be validated in a broader CA population.

Although EEGs were mandatory in the TTM-trial, examinations were still subject to selection bias, because patients awake or dead prior to the time point of prognostication were excluded from this study. Our patient population had a higher rate of poor outcome than the patients who were excluded, as patients who awoke prior to examination were excluded.^7,10^ We do not consider this a limitation because neurologic prognostication is only relevant in this group of comatose patients.

Small sample sizes in some subgroups resulted in limited power in subgroup analyses, which may be regarded as hypothesis-generating. The cohort included in this study was previously evaluated when validating highly malignant patterns as reported previously.^7,10^ EEG reactivity was not included in this study as not all collaborating sites performed reactivity testing. The ACNS criteria have been updated after the analysis performed in this study.^45^ However, ACNS definitions used in our study have undergone only minor changes, which we consider insignificant for the interpretation of our results.

The EEGs were performed >36 hours postarrest and we assume that our results could differ in EEGs performed earlier. Our findings need to be validated for EEGs performed <36 hours postarrest. We cannot exclude that patients had electrographic seizures, potentially affecting NfL levels, not detected by routine EEG, which might have been detected with continuous EEG monitoring. The rate of patients with ongoing antiseizure medication was higher with increasing amounts of discharges on the EEG. Nonetheless, 16% of patients without electrographic discharges were also treated with anticonvulsant drugs and 39% of patients had ongoing sedation during the EEG recording. It is possible that if left untreated, some patients would otherwise have demonstrated electrographic discharges, which may have influenced our results. We previously reported that the prognostic ability of EEG on the group level in the TTM-trial cohort was not significantly affected by ongoing sedation, but acknowledge that individual false-positive cases may occur; for instance, the good outcome patient with burst-suppression during significant sedation described above.^7,10^

The only prespecified EEG criterion allowing WLST in the TTM-trial was a therapy-refractory status epilepticus ≥108 hours postarrest.^19,44^ Results of local EEG reviews were available to treating physicians and therefore we cannot exclude the risk of a self-fulfilling prophecy on neurologic outcome. In contrast, NfL was analyzed after trial completion, thus levels were not available upon clinical decision-making, minimizing risk for bias. NfL has been validated for clinical use in some European countries but is not yet widely clinically available and there is no reference standard or international normal reference limit for serum NfL.^46^

EEG patterns >36 hours after cardiac arrest reflect the extent of brain injury as measured by NfL in serum. The EEG background is more strongly related to the extent of brain injury compared with superimposed discharges. A clinical scenario with unexpected combinations of high NfL levels with a continuous EEG pattern >36 hours could help identify patients with potentially poor outcome, and low NfL levels in patients with malignant or highly malignant patterns could help identify patients with potentially favorable outcome.

## Glossary

ACNS: American Clinical Neurophysiology Society
ANOVA: analysis of variance
CA: cardiac arrest
CPC: Cerebral Performance Category
NfL: neurofilament light
NSE: neuron-specific enolase
ROSC: return of spontaneous circulation
TTM-trial: Target Temperature Management After Out-of-Hospital Cardiac Arrest trial
WLST: withdrawal of life-sustaining therapy
